# Ketamine cystitis following ketamine therapy for treatment-resistant depression – case report

**DOI:** 10.1186/s12888-023-05468-3

**Published:** 2024-01-02

**Authors:** Minna Chang, Mario F Juruena, Allan H Young

**Affiliations:** 1 Epsom and St Helier Hospital University and Hospital Trust, Epsom Surrey, KT18 7EG UK; 2 Department of Psychological Medicine, Institute of Psychiatry, Psychology and Neuroscience, King’s College London, London, UK; 3 South London and Maudsley (SLaM) NHS Foundation Trust, London, UK; 4 Bethlem Royal Hospital, Monks Orchard Road, Beckenham, Kent BR3 3BX UK

**Keywords:** Ketamine, Treatment-resistant depression, Cystitis, KIC, Lower urinary tract symptoms

## Abstract

**Background:**

Ketamine is a novel and exciting putative antidepressant medication for patients with treatment-resistant depression. A complication commonly seen in frequent and heavy recreational use of ketamine is ulcerative cystitis, which presents with lower urinary tract symptoms (LUTS) and upper renal tract damage and can be seen in over 25% of regular users. Although Ketamine-induced cystitis (KIC) is a recognised complication in recreational use of ketamine, its occurrence in therapeutic use of ketamine in depression has so far not been reported. The exact pathogenesis of KIC is currently unknown, making treatment and prevention advice much more difficult. Early diagnosis of KIC and immediate cessation of ketamine has been shown to improve adverse urinary tract symptoms and prevent further damage.

**Case presentation:**

We present a case of a 28-year-old female who was started on ketamine treatment for depression, and who then developed symptoms of KIC, which was confirmed by urine microscopy, culture and analysis.

**Conclusions:**

To our knowledge, this is the first reported case of KIC in a patient receiving treatment-dose ketamine as part of their antidepressant therapy.

## Background

Ketamine has been shown to be an effective antidepressant in patients with treatment-resistant depression (TRD) with up to 71% positive response rate in these cohorts [1]. It acts as an N-methyl-D-aspartate (NMDA) receptor antagonist with glutamate blocking capacity with an onset of action much faster than conventional antidepressant medications [1, 2]. Depending on the mode of delivery, the antidepressant effects from ketamine administration can be seen within minutes to hours, and the benefits can last for days to weeks [1, 2].

Common symptoms of ketamine-induced cystitis (KIC) include urinary urgency, frequency and dysuria, progressing to incontinence, haematuria, ulcerative cystitis, hydronephrosis, bladder wall fibrosis and chronic kidney failure [3–6].

Regular ketamine use is associated with an increase in lower urinary tract symptoms (LUTS) by up to 3–4 times compared to healthy individuals. Urinary symptoms can be seen in over 25% of recreational users of ketamine, which is directly correlated with dose and frequency of use [3]. If ketamine cessation occurs early, the urinary symptoms can improve and early damage can be reversed [6].

We present a case of a 28-year-old female who was started on ketamine treatment for depression, and who developed symptoms of cystitis, which was confirmed by urine microscopy, culture and analysis. To our knowledge, this is the first reported case of KIC in a patient receiving treatment-dose ketamine as part of their antidepressant therapy.

## Case presentation

A 28-year-old female presented to the National Affective Disorders Service/Maudsley Advanced Treatment Service (NADS/MATS) at Maudsley Hospital with a relapse of her TRD. She had a past psychiatric history of severe unipolar TRD, which had previously been successfully treated with electroconvulsive therapy (ECT) and medications. Her past medical history also included epilepsy, for which she was taking sodium valproate 600 mg with good effect.

She was already on a combination of antidepressant medications, mood stabilizers and an antipsychotic. Her medication regime included: Vortioxetine 20 mg, Lithium (Priadel) 800 mg, Valproate 600 mg, Quetiapine 500 mg and Levothyroxine 150mcg.

Up until this relapse, her mood had been stable, and she reported taking her medications as prescribed.

On assessment, she was well-dressed and well-groomed. She spoke slowly and monotonously with one-syllable words and appeared dysthymic in nature. On discussion, it was evident that she had negativistic cognitive distortions and ideas of worthlessness. She was at times not fully able to engage in the assessment but was generally coherent. There were no signs of hallucinations or abnormal sensory experiences, but she did appear more depressed than previously and more easily distracted.

Due to this relapse, ketamine augmentation therapy was started on 25/10/2021 in the form of sublingual lozenges 160 mg twice weekly. This was increased to three times a week on 10/01/2022 with good effect. However, she complained of nausea and vomiting from the sublingual preparation.

In September 2022, her depression began to decline again, and on 27/09/2022 the treatment was switched to oral capsules 240 mg and escalated to four times a week. She reported a significant improvement in the nausea and vomiting following this switch.

The ketamine treatment showed an excellent antidepressant response. However, she began to complain of symptoms of dysuria which initially began insidiously. She described this as “stinging during and after peeing”. This discomfort would occasionally last for a few hours after voiding. These usually happened 12–24 h after taking the ketamine dose. She was managing the pain with over-the-counter paracetamol and phenazopyridine hydrochloride, which she found very helpful. She was advised to stay well hydrated whenever taking the ketamine and to continue monitoring her symptoms closely. She reported taking the ketamine exactly as prescribed and denied taking ketamine recreationally. Further examination of lower urinary tract symptoms (LUTS) in accordance with the International Continence Society (ICS) demonstrated no history of storage symptoms, voiding symptoms or post-micturition symptoms prior to the ketamine treatment.

Unfortunately, the frequency and severity of the dysuria progressively worsened. A urine microscopy, culture and stain demonstrated sterile pyuria, with positive inflammatory cells, but no growths, nitrites or blood, ruling out a urinary tract infection (UTI). As such, the patient’s symptoms of urgency and dysuria were attributed to the ketamine use. It is possible that these symptoms could have been idiopathic and unrelated to the ketamine. However, given the temporal relationship between starting the ketamine and the development of the symptoms, and the alleviation of symptoms on discontinuing the ketamine, in the absence of cystoscopic evaluation, the association was assumed to be causal.

There were no indications of a UTI or a sexually transmitted infection. No intimate examinations, including cystoscopy, were performed.

Since the patient’s urinary symptoms were detected early and the ketamine was subsequently withdrawn early, her bladder and renal tract symptoms did not progress to such a degree that she needed to be referred to urology for more advanced and invasive investigations and treatment.

Blood tests were unremarkable and renal function was normal. This included: Na 141mmol/L, K 4.7mmol/L, urea 3.2mmol/L, creatinine 80mmol/L, GFR 89, WCC 5.2 × 109/L.

Due to the concerns about irreversible bladder and renal tract injury, the decision was made to withdraw the ketamine. The patient reported that within 3 weeks, the symptoms of dysuria had completely resolved, but her mood had worsened. A repeat urinalysis carried out at this point yielded normal results. A decision was made to begin ECT and to rationalise her medications, which included discontinuing the ketamine.

## Patient perspective

“The ketamine was really helpful for my mood – it worked quickly and my family, friends and I all noticed the difference straight away. My mood was better, I had more energy and motivation to do things, my thoughts took a more positive swing and I was actually genuinely happy to be alive again. Sadly, the urinary symptoms were horrible – they felt like a really bad urinary tract infection and the pain lingered longer each time. In the end, the urinary symptoms made me start to dread each dose of ketamine, as I knew that the pain would be there for most of the day and night afterwards. As a result, making the decision to stop the ketamine was a difficult one, but so was the idea of continuing it”.

## Discussion

Over 350 million people worldwide suffer from depression and about one-third of these patients are believed to have treatment-resistant depression (TRD) [7]. TRD is most commonly defined as failure to two or more adequate trials of antidepressant medications [8].

Ketamine has been shown to be a novel and exciting putative antidepressant in patients with TRD, with up to 71% positive response rate [1]. It acts as an N-methyl-D-aspartate (NMDA) receptor antagonist with glutamate-blocking capacity and has an onset of action much faster than conventional antidepressant medications [1, 2]. Depending on the mode of delivery, the anti-depressant effects can be seen within minutes to hours and the benefits can last from days to weeks [1, 2].

Ketamine-induced cystitis (KIC) typically starts with urinary symptoms that include dysuria, urgency, nocturia and urinary frequency [3, 5]. With continued use, symptoms can progress to incontinence, haematuria, bladder wall fibrosis and ulcerative cystitis. Ongoing use of the drug can lead to involvement of the upper renal tract, including hydronephrosis and chronic kidney failure [4–6]. Physical examination and investigations may demonstration suprapubic pain, sterile pyuria and increased eosinophils within the bladder wall [6]. The pathophysiological mechanisms of LUTS are not yet fully understood and further research to define therapeutic options would be useful [9].

Imaging of the bladder in severe cases may demonstration a grossly constricted bladder with thickened walls [6]. Cystoscopy often demonstrates a friable bladder mucosa that is prone to bleeding [6]. Microscopically, the urothelium may appear denuded, ulcerated and infiltrated by inflammatory cells, such as mast cells and eosinophils. Other findings include submucosal fibrosis, muscle hypertrophy and collagen deposition [10].

Although the exact pathogenesis of KIC is not yet fully understood, various mechanisms have been postulated and it is likely that several pathways are involved simultaneously [11].

One theory is that the ketamine and its metabolites (which are largely excreted by the urinary tract), cause direct toxicity to the bladder. These disrupt the urothelial integrity of the bladder epithelium and initiate interstitial fibrosis. This has been demonstrated in animal models and the level of damage directly correlates with the dose of ketamine used [12].

Another theory is an IgE-mediated response. Bladder samples in ketamine users frequently show raised inflammatory cells and messengers, including mast cells, eosinophils, COX-2 (cyclo-oxygynase-2), NOS (nitric oxide synthase) and IgE. These levels fall once the patient is in remission from ketamine use and rise again once ketamine is restarted. This suggests an inflammatory response or a hypersensitivity reaction leading to bladder damage [6, 12].

Ketamine can also directly stimulate various chemicals, including adenosine triphosphate, antiproliferative factor and oxidative stressors, which subsequently lead to changes in the bladder wall [6]. It has been reported that the NMDA receptor (NMDAR) and angiogenic factors can also cause microvascular injury within the bladder [6].

Other proposed theories include aberrant neurotrophic factors, protein kinase B, mTOR pathways, metadherin and MAPK pathways, leading to downstream fibrosis of the bladder [6].

Early diagnosis of KIC and immediate cessation of ketamine use has been shown to improve symptoms, reverse early disease and prevent further damage [6].

Spravato (esketamine) is currently the licensed treatment for treatment-resistant depression. A number of phase III trials have compared the incidence of urinary tract-related complications in patients receiving esketamine in comparison to placebo. Several studies have reported no new incidences of cystitis in adult patients treated with esketamine [13–15]. However, the rate of LUTS has been reported to be higher in these patients [16]. Long-term administration of esketamine for up to 4.5 years (SUSTAIN-3 study), has been associated with LUTS, including dysuria (2.7%), pollakiuria (2.4%), urgency (1.3%), nephrolithiasis (1.3%), haematuria (1.0%) and incontinence (1.0%) [13]. However, direct analysis of urine samples from patients on regular esketamine treatment showed no significant changes in urinary leukocyte, erythrocyte, haemoglobin or protein concentrations, indicating that urothelial toxicity and interstitial cystitis was unlikely in this cohort [17].

## Conclusions

To our knowledge, this is the first reported case of ketamine-induced cystitis (KIC) in patients receiving treatment-dose ketamine as part of their antidepressant therapy. Our case highlights the importance of actively monitoring for symptoms of lower urinary tract symptoms and KIC in all patients taking ketamine for depression, especially now that the use of this therapy is rapidly rising globally.

Further research is required to determine the safe frequency, dose, route and duration of ketamine as an antidepressant therapy to avoid KIC. We also advise further research to identify individual risk factors that may play a role in determining the susceptibility and likelihood of developing KIC. A greater understanding of the mechanisms by which esketamine is able to limit its toxic effects on the bladder would be useful. Furthermore, we recommend regular screening for urinary symptoms in all patients receiving ketamine treatment.

## Data Availability

Not applicable.

## List of abbreviations

COX-2: Cyclo–oxygynase–2
ECT: Electroconvulsive Therapy
ICS: International Continence Society
KIC: Ketamine–induced Cystitis
LUTS: Lower Urinary Tract Symptoms
NMDA: N–methyl–D–aspartate
NMDAR: N–methyl–D–aspartate receptor
NOS: Nitric Oxide Synthase
TRD: Treatment–resistant Depression
UTI: Urinary Tract Infection
